# Impact and effect of imaging referral guidelines on patients and radiology services: a systematic review

**DOI:** 10.1007/s00330-024-10938-7

**Published:** 2024-07-13

**Authors:** Yi Xiang Tay, Shane Foley, Ronan Killeen, Marcus E. H. Ong, Robert Chun Chen, Lai Peng Chan, May San Mak, Jonathan P. McNulty

**Affiliations:** 1 Radiography and Diagnostic Imaging, School of Medicine, University College Dublin, Dublin, Ireland; 2 Radiography Department, Allied Health Division, Singapore General Hospital, Singapore, Singapore; 3 St Vincent’s University Hospital, Dublin, Ireland; 4 School of Medicine, University College Dublin, Dublin, Ireland; 5 Department of Emergency Medicine, Division of Medicine, Singapore General Hospital, Singapore, Singapore; 6 Duke-NUS Graduate Medical School, Singapore, Singapore; 7 Department of Neuroradiology, Division of Radiological Sciences, Singapore General Hospital, Singapore, Singapore; 8 National Neuroscience Institute, Singapore, Singapore; 9 Department of Diagnostic Radiology, Division of Radiological Sciences, Singapore General Hospital, Singapore, Singapore

**Keywords:** Value-based healthcare, Clinical decision support, Radiology, Evidence-based practice

## Abstract

**Objectives:**

The objective of this systematic review was to offer a comprehensive overview and explore the associated outcomes from imaging referral guidelines on various key stakeholders, such as patients and radiologists.

**Materials and methods:**

An electronic database search was conducted in Medline, Embase and Web of Science to retrieve citations published between 2013 and 2023. The search was constructed using medical subject headings and keywords. Only full-text articles and reviews written in English were included. The quality of the included papers was assessed using the mixed methods appraisal tool. A narrative synthesis was undertaken for the selected articles.

**Results:**

The search yielded 4384 records. Following the abstract, full-text screening, and removal of duplication, 31 studies of varying levels of quality were included in the final analysis. Imaging referral guidelines from the American College of Radiology were most commonly used. Clinical decision support systems were the most evaluated mode of intervention, either integrated or standalone. Interventions showed reduced patient radiation doses and waiting times for imaging. There was a general reduction in radiology workload and utilisation of diagnostic imaging. Low-value imaging utilisation decreased with an increase in the appropriateness of imaging referrals and ratings and cost savings. Clinical effectiveness was maintained during the intervention period without notable adverse consequences.

**Conclusion:**

Using evidence-based imaging referral guidelines improves the quality of healthcare and outcomes while reducing healthcare costs. Imaging referral guidelines are one essential component of improving the value of radiology in the healthcare system.

**Clinical relevance statement:**

There is a need for broader dissemination of imaging referral guidelines to healthcare providers globally in tandem with the harmonisation of the application of these guidelines to improve the overall value of radiology within the healthcare system.

**Key Points:**

- *The application of imaging referral guidelines has an impact and effect on patients, radiologists, and health policymakers*.
- *The adoption of imaging referral guidelines in clinical practice can impact healthcare costs and improve healthcare quality and outcomes*.
- *Implementing imaging referral guidelines contributes to the attainment of value-based radiology*.

## Introduction

Value-based healthcare is a “transformative framework” that alters the delivery of care by healthcare professionals and healthcare institutions [1]. It offers the potential to provide care in a cost-effective manner while improving patient health outcomes through the optimisation of available finite resources. The concept of low-value care can be described as “the use of a health service for which the harms or costs outweigh the benefits” [2]. This practice contradicts the objectives of value-based healthcare but is still widely prevalent [3–8]. This underscores the need for a worldwide effort to mitigate it [9].

Diagnostic imaging has been recognised as a significant factor contributing to the inefficient use of limited healthcare resources on a global scale. A recent systematic review revealed that there exists a range of low-value imaging procedures, varying from 2% to ≥ 90%, for both adult and paediatric populations [10]. The utilisation of medical imaging plays a crucial role in determining patient outcomes, but the excessive use of low-value imaging procedures results in an inefficient allocation of resources and poses potential dangers [11–13]. Indeed, the continued growth in the frequency of radiology use can lead to resultant knock-on effects on healthcare resources and spending; therefore, focused efforts are required to curb these unsustainable continual increases in imaging [14].

Several international radiology societies, including the American College of Radiology (ACR), the European Society of Radiology (ESR) and the Royal College of Radiologists (RCR), have long acknowledged the importance of incorporating evidence-based imaging referral guidelines into clinical practice. However, the extent of progress in implementing these guidelines as a form of decision-aiding tool has varied across different regions worldwide [15–17]. These measures have raised optimism over the reduction of low-value imaging, leading to greater appropriateness in referrals and a decrease in unnecessary radiation exposure [15]. Nevertheless, recent evidence suggests that the appropriateness of imaging is still lacking across the world [18, 19], and this contradicts the attainment of value-based radiology (VBR) [20].

Over time, there has been a significant increase in global endorsement for evidence-based imaging referral guidelines, leading several governments to contemplate the adoption or adaptation of existing imaging referral guidelines [21]. Recent scoping reviews have brought attention to the examination of imaging referral guidelines that are employed to establish and evaluate the appropriateness or value of diagnostic imaging [10, 22]. However, a comprehensive systematic review assessing the impact and effect of imaging referral guidelines on VBR is currently lacking. Knowledge about the impact and effect could potentially advance the implementation of imaging referral guidelines as a strategic thrust for VBR. Hence, the objective of this systematic review was to provide an overview and explore the associated outcomes of imaging referral guidelines for key stakeholders including patients and radiologists.

## Materials and methods

This review was conducted in accordance with the preferred reporting items for systematic reviews and meta-analyses guidelines (PRISMA) [23]. The protocol for this systematic review is prospectively registered on the PROSPERO website (CRD42023420023). [24] The databases Embase, MEDLINE and Web of Science were searched for studies published from January 2013 to May 8, 2023.

Search terms were used with Boolean operators in EMBASE and adapted for the other databases. The search terms used are reported in Table 1.

**Table 1:** Search terms combined with the Boolean operators

(‘radiology’ OR ‘service*‘ OR ‘department’ OR ‘radiography’ OR ‘X-ray’ OR ‘computed tomography’ OR ‘ct’ OR ‘tomography’ OR ‘ultraso* ‘OR ‘mri’ OR ‘magnetic resonance imaging’ OR ‘mammography’ OR ‘nuclear medicine’ OR ‘fluoroscopy’ OR ‘diagnostic imaging’ OR ‘medical imaging’ OR ‘patient*’) AND
(‘referral guidelines’ OR ‘referral criteria’ OR ‘referral recommendations’ OR ‘appropriateness criteria/exp’ OR ‘appropriateness criteria’ OR ‘choose wisely’ OR ‘radiological guidelines’) AND
(‘impact*’ OR ‘effect*’ OR ‘improv*’ OR ‘reduc*’ OR ‘increase*’ OR ‘decrease*’ OR ‘saving’ OR ‘outcome*’ OR ‘utilisation’ OR ‘utilization’ OR ‘cost*’ OR ‘finance*’ OR ‘dose*’ OR ‘value*’ OR ‘low*’ OR ‘high*’ OR ‘sustainability’ OR ‘time*’ OR ‘appropriate*’ OR ‘radiation protection’)

The records from the databases were exported and archived in a Microsoft Excel spreadsheet (Microsoft Corporation). Titles and abstracts were screened based on inclusion and exclusion criteria (Table 2), with duplicates removed.

**Table 2:** Inclusion and exclusion criteria

Inclusion criteria	English language publication
	Publication format—article, article in press and reviews
	Implementation of imaging referral guidelines
	Impact/effect on patient or radiology services
Exclusion criteria	Published in languages other than English
	Commentary, editorials, conference papers and proceedings
	Animal studies

Y.X.T. performed the data extraction from the selected articles, and the extracted data was cross-checked by J.P.M. and S.F. The mixed methods appraisal tool (MMAT) was used to appraise the methodological quality of the selected studies. MMAT is well suited for the appraisal stage of this review as it facilitates critical appraisal of the most common types of study methodologies and designs [25].

## Results

### Literature search

**Fig. 1:**
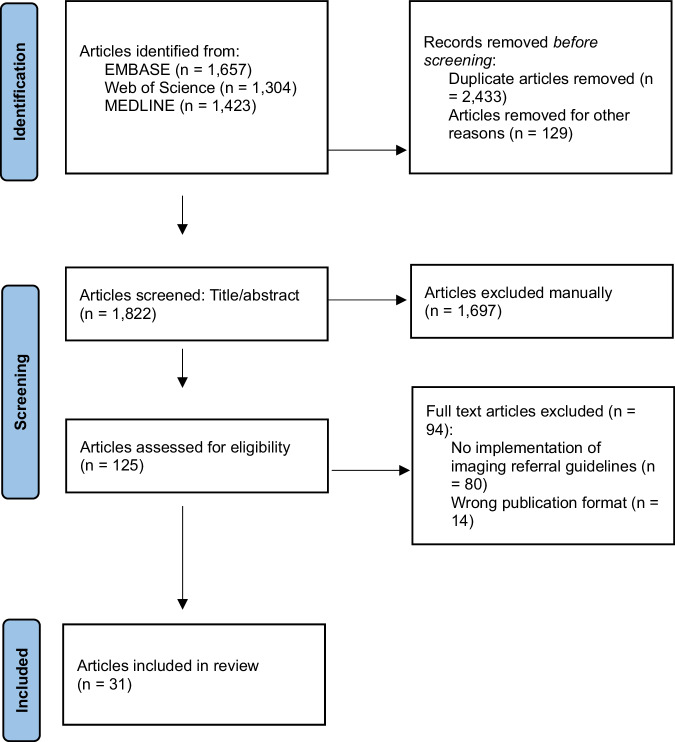
PRISMA flow diagram of the screening and selection process

The three electronic database searches yielded 4384 records—EMBASE: 1657; MEDLINE: 1423 and Web of Science: 1304. A total of 2433 records were removed for duplication and another 129 were removed for language and format of publication. The remaining 1822 records were screened for title and abstract which excluded 1697 records. Following full-text screening of 125 articles, 94 articles were excluded; an overview of the excluded articles and the reason for exclusion is presented in Supplemental Table 1. Ultimately, 31 studies were included in this narrative synthesis. A PRISMA flow diagram of the screening and selection process is presented in Fig. 1.

### Quality and risk of bias assessment

Based on MMAT, studies were divided into several study design categories and evaluated using the respective methodological quality criteria [25]. Ten studies were found to satisfy all the specified criteria (these are marked with a * in Supplemental Table 2), whilst the remaining papers either failed to meet one of the criteria or lacked adequate information in their respective reports. The full MMAT report is available in Supplemental Table 2.

### Study characteristics

The characteristics of the studies that were included were summarised in Supplemental Table 3. The ACR Appropriateness Criteria^®^ (*n* = 18) were the most commonly reviewed imaging referral guidelines by radiology societies. This was followed by ESR iGuide (*n* = 1) and RCR iRefer (*n* = 1). There were also other studies that looked at non-radiological societies’ imaging referral recommendations. The two most commonly cited recommendations were Choosing Wisely (*n* = 5) and European Commission Radiation Protection 118; referral guidelines for imaging (*n* = 3).

Studies were undertaken in a total of nine different countries, with the majority of the studies from the United States (*n* = 17) and Europe (*n* = 6). In-hospital imaging was the most common clinical setting (*n* = 19/29; 66%) and most studies involved multi-institutions (*n* = 17; 59%). Nine studies included adult outpatients, five studies included patients from the emergency department, two studied paediatric population in the emergency department and three studied in-patients. The most commonly reported imaging modalities were CT, MRI and conventional radiography.

Outcomes measured for the impact and effect of imaging referral guidelines (intervention) varied across the studies and the most common was the volume of imaging (*n* = 16), portion of appropriate/high-value imaging (*n* = 15) and appropriateness rating/decision support score of imaging (*n* = 9).

### Reported findings

There exists a myriad of approaches for the implementation of interventions. These include the implementation of active education and imaging referral guidelines [26–33], utilisation of checklists [34], dissemination of imaging referral guidelines [35, 36], implementation of an integrated clinical decision support system (CDSS) [37–46], and the development of application-based imaging referral guidelines [47, 48]. Collaboration and communication programs including radiology support, communication and alignment network (R-SCAN) initiatives [13, 49–51] and multifaceted campaigns [27, 52, 53] were also approaches adopted for the implementation of imaging referral guidelines.

The utilisation of meta-analysis was deemed unsuitable for this corpus of literature due to the extensive heterogeneity observed between studies with regard to research design, study population, interventions employed, and outcomes assessed. As such, it is not possible to obtain a useful overall estimate of the effect. Therefore, a narrative synthesis was conducted in order to amalgamate the findings from various studies.

In the following sections, findings are stratified by the perspectives of stakeholders involved in VBR (patients, radiologists, and health policymakers). The key findings of the studies were summarised in Supplemental Table 4.

### Patients

The impact and effect of imaging referral guidelines on patients were explored in five studies [29, 31, 33, 34, 51]. Specifically, the implementation of imaging referral guidelines was reported to reduce patients’ radiation exposure/dose from CT and conventional radiography [29, 31, 33]. Covington et al documented a 16% decrease in the average patient radiation dose from medical imaging from 16.7 mSv to 14.0 mSv [31]. Similarly, in a study conducted by Tahvonen et al, it was observed that there was a reduction of 52% in the collective effective dose associated with spine radiography over a period of six months [29]. Overall, the implementation of imaging referral guidelines has resulted in a reduction in radiation exposure while also reducing unnecessary intravenous contrast administration, mitigating the potential deleterious effects associated with contrast agents [33].

Xu et al reported on the topic of patient wait times, an area of significant focus [34]. The average wait times for an MRI of the knee showed a decrease, from 23.3 ± 9.1 days prior to implementation to 17.4 ± 5.3 days following implementation of an appropriateness checklist. Although Wang et al did not provide a specific analysis of patient wait times, they did observe a significant increase in the duration (13.6–21.6 weeks) between a patient’s initial clinic visit for uncomplicated lower back pain and their getting a lumbar spine MRI following the intervention of R-SCAN programme [51]. Covington et al concluded that patient satisfaction remained consistent during the education intervention on appropriate abdominal imaging, with a positive evaluation of overall medical care maintained at 69% and 68% over the course of the two-year study period [31].

### Radiologists

The 14 studies included in the review provided information on various parameters related to workload and utilisation of diagnostic imaging. A total of seven studies documented a decrease in the volume of imaging referrals, primarily for CT and CR imaging [26, 27, 29, 33, 35, 47, 51]. There was a statistically significant decrease in imaging volume demonstrated across the multiple studies. No significant changes in the volume/rate of imaging referrals were reported by three studies, albeit a 12% increase in the total number of CT imaging performed over the 5-year study period as found by Tahvonen et al [28, 31, 39]. In fact, Covington et al also found that there was no significant difference (*p* = 0.45) in radiation-free imaging (ultrasound and MRI) performed per patient after a reduction in mean abdominal CT imaging following an educational intervention that incorporated the ACR appropriateness criteria [31].

A similar trend was noted when examining the mean number/ number of referrals per month. Five studies [30, 33, 35, 47, 51] revealed a decrease in the mean number of referrals per month. Covington et al further reported a decrease in the total number of abdominal imaging per patient including abdominal CT, with no significant differences in the use of non-ionising imaging modalities (MRI and US) [31]. In the included studies, three studies reported no significant differences post-intervention of imaging referral guidelines. However, Palen et al observed in their randomised controlled trial (RCT) that, although there were no significant changes in the monthly rate of imaging referral following the intervention of a CDSS, the mean number of imaging referrals increased from 10.85 per member-month to 11.14 after intervention [43].

In terms of workload related to changes in imaging referrals made by radiologists to clinicians, a decrease of 0.4% was reported by Palen et al in their RCT evaluating the use of imaging referral guidelines embedded in a CDSS [43]. Furthermore, Moriarity et al showed a notable reduction in the number of referrals with insufficient structured clinical information at the point of order entry, facilitating appropriateness scoring by CDSS. This effect was particularly pronounced among primary care practitioners in comparison to specialists [39].

### Health policymakers

A total of four studies reported the impact and effect of CDSS, R-SCAN programme and campaign on high-value imaging [13, 40, 49, 53]. In a single institutional study by Huber et al [40], high-value imaging increased from 64.5% to 82%, with an increase noted in imaging modalities such as CT, MRI and US, albeit with a 14.1% increase in low-value PET and NM imaging. Studies involving multiple other institutions show a similar trend. Rezaii et al [49] reported from their study involving 27 practices an increase of high-value imaging from 57% to 79%, whereas Wintermark et al [13] observed an increase in high-value imaging ranging from 12% to 37% across CT and MRI imaging.

The appropriateness of imaging referral following intervention was reported in nine studies [26, 28, 29, 38, 39, 44, 46, 48, 52]. All studies conducted reported a notable rise in the number of appropriate imaging referrals over a varying period of one month to three years, accompanied by a corresponding downward trend in inappropriate imaging referrals. The aforementioned trend was sustained over time and was documented by Tahvonen et al [26] in their longitudinal study spanning three years. The study found that the level of appropriateness was not subject to any significant changes during the follow-up period. A total of seven studies explored the appropriateness rating/score after the intervention, with the majority indicating a positive change in the appropriateness rating/score [38, 39, 43, 50–52]. Only one study [33] revealed no significant difference following an education initiative and the findings of the study align with the literature review conducted by Bhattacharyya et al [54], which indicated that the publication of appropriateness criteria has not universally resulted in improvements in the appropriateness of imaging. Several studies reported a range of 2.0–13.8% for the proportion of low appropriateness rating/score [37, 40–42] and a range of 27–82% for the portion of high appropriateness rating/score [40–42, 45].

The intervention’s cost-saving significance was reported in four studies. Two of these studies [31, 33] reported on the cost savings associated with head and abdominal CT; another reported [13] on CT/MRI imaging (CTA for pulmonary embolism, advanced imaging for low back pain and adnexal cyst imaging) and the remaining study [35] examined the cost savings related to myocardial perfusion imaging. Through the implementation of education initiatives, Strother et al [33] observed an estimated reduction in healthcare costs of US$ 117,000 per year, whereas Covington et al [31] and Wintermark et al [13] observed a total saving of US$ 81,528 and US$ 257,844, respectively. Roifman et al [35] also observed a notable decrease in costs amounting to CAN$ 72,056,539 as a result of the reduced utilisation rate of myocardial perfusion imaging following the publication of appropriate use criteria in Ontario Province, Canada.

The studies included also addressed the domain of clinical effectiveness. The utilisation of the appropriateness checklist in knee MR imaging resulted in a reduction in the frequency of radiological outcomes indicative of moderate or more severe osteoarthritis, therefore mitigating the occurrence of wasteful studies [34]. In the context of trauma, an education initiative on the appropriate utilisation of screening cervical spine CT resulted in the enhanced clinical effectiveness of imaging, leading to a considerable increase in the detection rate of cervical spine injury from 1.0% to 3.1% (*p* = 0.022) in the emergency department [32]. The clinical efficacy of low-value dual-phase head CT was shown to be poor, as this imaging technique provided less than 1% additional diagnostic information for screening or managing suspected or known brain metastases [33]. These findings were consistent with the study conducted by Gupta et al on CDSS, which indicated that low-value imaging requested by patients yielded lower rates of positive imaging outcomes for sinus CT (27%), spine CT/MRI (10.5%), and imaging for the evaluation of an abdominal aortic aneurysm (4%) [37].

The studies also revealed additional findings, such as an elevated degree of understanding regarding radiation protection and clinical imaging guidelines following educational initiatives targeting the use of imaging referral guidelines [48]. Second-year medical students who participated in such an educational initiative that emphasised appropriateness criteria exhibited enhanced knowledge of the appropriate use of medical imaging and demonstrated increased awareness of radiation protection [55]. At the same time, there were no significant differences observed in the clinical load of downstream services, such as physical therapy, after implementing R-SCAN [50, 51]. Likewise, after the implementation of an education initiative, there was no significant difference observed in terms of in-hospital mortality rates or the average duration of patient stays [31].

For the clinicians, the benefits of imaging referral guidelines were compelling. Surveys and interviews indicated that interventions, such as Choosing Wisely, had empowered them positively in their clinical decision-making regarding imaging referrals [53]. Additionally, it enhanced their dialogue with patients about low-value services [53]. Thematic analysis from focus group discussions involving neurology and neurosurgery residents also revealed that clinicians prioritise functionality, usefulness and effectiveness when using imaging referral guidelines in their clinical practice [47].

## Discussion

The findings of this systematic review demonstrate the immediate and extended positive impacts and effects of imaging referral guidelines, encompassing outcomes that were centred on value, patient and service provision.

During this review, the efficacy of CDSS in reducing overutilisation and its associated outcomes were extensively identified. Some of these systems are integrated with the electronic health record system, while others operate as standalone models. These integrated systems offer valuable guidelines for physicians at all levels of training and with different levels of knowledge regarding imaging referral guidelines [38]. The results of our review were consistent with Sutton et al [56] comprehensive study looking at the utilisation of CDSS in the field of medicine. The advantages of CDSS were broadly classified into four categories: enhancing patient safety, improving clinical management, containing costs, and enhancing workflow efficiency.

However, CDSS possesses intrinsic limitations. Several studies examining CDSS have indicated that there is an absence of guidelines for numerous clinical conditions, leading to substantial unrated imaging referrals [42, 44]. There was also apprehension over clinicians gaming the CDSS in order to produce high CDS or indicated scores [40, 45]. Additionally, current CDSS have been found to be incapable of doing natural language processing to extract valuable data from unstructured free-text clinical information provided by physicians [39, 40, 44, 45]. These observations give rise to a sense of uncertainty over the actual efficacy of CDSS in enhancing quality or diminishing costs. Furthermore, given the limited number of studies that have conducted economic evaluations of CDSS, justification of the additional costs of this supplementary system may be warranted.

One crucial factor that promotes the utilisation of guidelines is their convenient accessibility [57]. In this context, standalone CDSS models facilitate imaging referral guidelines to be accessed either through digital means on mobile devices or workstations. Furthermore, these models operate independently, alleviating the complexities associated with information technology infrastructure and the need for system integration. Nevertheless, it is important to acknowledge that the utilisation of application-based imaging referral guidelines is not without its limits. One such drawback is that the review of these guidelines is just discretionary, which may result in a lack of sustained and enduring change [47]. Another challenge that arises is the sustainability of long-term utilisation and its relevance to daily clinical practice [54]. This is due to the additional time involvement associated with using the application, which may lead to the discontinuation of its use by many individuals [48]. Furthermore, the use of standalone models is not recommended due to the need for duplicating data entry and the difficulties in tracking data across several groups and individuals, hence preventing data-driven improvement [58, 59]. After all, an individual feedback loop would be crucial to sustain changes in imaging referral behaviour [59].

Non-technological methods of intervention have also demonstrated the ability to enhance the utility of imaging while simultaneously contributing to cost savings in healthcare. Covington et al [31] implemented a cost-effective educational intervention in the form of lectures at a community-based teaching hospital, focusing on appropriate abdominal imaging. This initiative, which had a minimal cost, yielded substantial cost savings and, in fact, demonstrated a reduction in the inappropriate radiological referral-ordering behaviour of internal medicine residents. This reduction was achieved by increasing knowledge regarding healthcare expenses and the excessive use of diagnostic imaging. As recently extorted, “outreach and medical education in imaging appropriateness guidelines, integrated at an early stage of training and continued throughout the course of one’s career,” this intervention is particularly well-suited due to its minimal cost and effectiveness in mitigating the issue of overutilisation of imaging [60]. However, it is important to note that sustaining a decrease in the utilisation of inappropriate imaging may necessitate the implementation of a continuous programme of ongoing education and individual feedback [54]. Indeed, the review of the evidence indicates that over a ten-year period, the proportion of appropriate cardiac imaging performed increased while the appropriateness rate for cardiac imaging remained unchanged [54]. This indicates that simply publishing imaging referral guidelines without implementing a comprehensive approach does not universally improve the appropriateness of imaging [54].

With VBR here to stay, radiology can greatly contribute to moving from a “volume-driven system to a value-driven one” [11]. The R-SCAN initiative serves as a model in which patients, clinicians, and radiologists work together to improve the appropriateness of diagnostic imaging, resulting in collaborative and patient-centred benefits [49, 50]. In this initiative, the collaborative effort between radiologists and referring clinicians was built upon a shared objective and commitment to reducing imaging inappropriateness, much like the common goal of value-based care transformation [51]. In fact, Wintermark et al [13] have reached the conclusion that their analysis of the impact of R-SCAN on healthcare imaging costs demonstrates the potential for significant cost reductions if implemented on a global scale for the Medicare demographic.

Health policymakers also have a significant role to play in this journey towards value-based healthcare. In a commentary by Mjåset et al [61] the significance of government involvement in care organisation as a crucial determinant for the successful implementation of value-based healthcare was highlighted. By removing institutional legacies and addressing competing interests among providers, it is possible to effectively navigate and manage complexity [61]. This will facilitate the healthcare system in generating greater value for patients, so ensuring that the pursuit of enhancing value in healthcare is not an unattainable “utopian ideal” [62]. Nevertheless, it is important to recognise that imaging referral guidelines have their limitations, as Gupta et al have observed, whereby positive imaging outcomes can still be detected in low-value imaging studies, improving healthcare outcomes, but many do not [37].

Furthermore, the existing imaging referral guidelines do not address several common clinical scenarios [63]. Similarly, the guidelines for paediatric imaging lacked coverage in two-thirds of clinical scenarios involving children [64]. Indeed, the lack of coverage in the guidelines, for instance, for patients with neoplasm, might result in cases classified as ‘Not included in the guideline’ instead of ‘Appropriate’ or ‘Inappropriate’. In such situations, it may be necessary to follow local protocols and consensus, or defer to expert opinion when there is a lack of evidence or ambiguity [65]. These underlying issues could have implications for systemic use.

There were also substantial numbers of imaging referral guidelines supported by expert opinion, whereas a smaller number were based on evidence from well-designed randomised controlled trials or high-quality meta-analyses [66]. According to Sardanelli et al [67], “strong evidence does not always produce a strong recommendation”; this notion can hinder the optimisation of the advantages provided by guidelines. The basis of expert opinion, together with the sum of evidence, can result in a variation of opinion. These differences may cause challenges in the acceptance of imaging referral guidelines by referring clinicians or even among radiologists [67].

Although the studies that were included did not specifically explore the effects of imaging referral guidelines on healthcare sustainability, there exists a potential for the radiology field to actively contribute to healthcare sustainability through this intervention. In their study, Alshqaqeeq et al [68] examined the application of ACR appropriateness criteria as an approach to mitigating hospital energy consumption and identified imaging modalities that have a reduced environmental impact while maintaining the same level of patient care quality. The findings of the study align with the conclusions reported by McAlister et al [69] who conducted a study into the carbon footprint associated with hospital diagnostic imaging in Australia. Their findings indicate that clinicians have the potential to reduce carbon emissions from diagnostic imaging through two primary strategies: firstly, by minimising the incidence of ordering unnecessary imaging procedures; and secondly, by selecting low-impact imaging modalities such as X-ray and ultrasound instead of high-impact options like MRI and CT, but only when it is clinically appropriate.

### Strengths and limitations

A strength of this review is that we exercised caution regarding potential reporting bias and so by not reviewing the reference lists of the selected articles in order to avoid citation bias. Additionally, we conducted searches across different databases, including Medline and non-Medline publications, to mitigate the possibility of location bias.

Nevertheless, it is important to note that the studies included in this analysis reflect a significant degree of heterogeneity in terms of their research design, study population, interventions utilised, and outcomes evaluated. The literature review exclusively included full-text articles, with a specific restriction on those published in English. This limitation may impact the interpretation of the overall effectiveness of the intervention, though most imaging referral guidelines are predominantly published in English. In addition, the potential for publication bias exists as only full-text articles are included. As a result of these constraints, there is a possibility that additional pertinent research may have been overlooked.

In the future, we recommend prioritising health economics analyses. Clearly, the costs of implementing imaging referral guidelines will vary by setting and specific method of implementation. Considerations of cost should include not only the cost incurred by the end user in utilising the imaging referral guidelines but also the full range of healthcare institutional costs. Costs from other sectors and productivity impacts should also be factored in. Similarly, the utilisation of imaging referral guidelines on social, psychological, and economical patient outcomes should be further explored as part of patient and public involvement and engagement. This could provide the profession with significant insights into these patients’ experiences, promoting collaboration among all stakeholders, fostering productive relationships and ensuring that imaging referral guidelines also effectively address patients’ needs [70].

Given the paucity of studies on imaging referral guidelines for follow-up imaging, additional studies are required to support the prescription of imaging referral guidelines in healthcare systems and establish their contribution to the overall management of the patient. This includes deriving indicators such as life expectancy and quality-adjusted life-years. Additionally, investigations on the effects of imaging referral guidelines on healthcare sustainability, i.e. carbon footprint, can be conducted as we embark on creating a greener, more sustainable healthcare system.

## Conclusion

In this review, there was convincing evidence that the adoption of evidence-based imaging referral guidelines in clinical practice can effectively impact healthcare costs and improve healthcare quality and outcomes, hence increasing the systemic value of radiology. As value-based healthcare proliferates in the healthcare landscape, the positive impact and effect of these imaging referral guidelines mean that interventions such as CDSS continue to hold considerable promise. However, the published papers illustrating the various forms of intervention have demonstrated varying efficacy. We anticipate that the result of this review will stimulate the radiological community to more rigorously evaluate the impact of imaging referral guidelines through robust prospective studies and to move towards harmonising the application of imaging referral guidelines while facilitating a broader dissemination of these guidelines to healthcare providers globally.

## Supplementary information

Supplementary material

## Abbreviations

ACR: American College of Radiology
CDSS: Clinical decision support system
ESR: European Society of Radiology
MMAT: Mixed methods appraisal tool
PRISMA: Preferred reporting items for systematic reviews and meta-analyses
RCR: Royal College of Radiologists
RCT: Randomised controlled trial
R-SCAN: Radiology support, communication and alignment network
VBR: Value-based radiology
