# Supplementary material for: Impact and effect of imaging referral guidelines on patients and radiology services: a systematic review
Source: Eur Radiol. 2024 Jul 13;35(1):532–41. doi: 10.1007/s00330-024-10938-7 (PMC11632068; doi:10.1007/s00330-024-10938-7)
Supplement: Supplementary file 1 — Supplementary material [file 330_2024_10938_MOESM1_ESM.pdf]

Supplemental Table 1: Excluded articles and reason(s) for exclusion		
Title	Author Names	Reason(s) for exclusion
ACR Select Identifies Inappropriate Underutilization of Magnetic Resonance Imaging in British Columbia	Eddy K et al. 2015	No implementation of imaging referral guidelines.
Adherence to the 2010 American College of Cardiology Foundation Appropriate Use Criteria for Cardiac Computed Tomography: Quality Analysis at a Tertiary Referral Center	Sidhu M.S et al. 2016	No implementation of imaging referral guidelines.
American College of Radiology (ACR) Appropriateness Criteria and EURO-2000 Guidelines Offer Limited Guidance for MRI Imaging of Pediatric Patients	Simoni P et al. 2022	No implementation of imaging referral guidelines.
American College of Radiology Appropriateness Criteria: Advancing Evidence-Based Imaging Practice	Subramaniam R.M et al. 2019	Commentary paper.<br><br>No implementation of imaging referral guidelines.
An audit of clinical practice, referral patterns, and appropriateness of clinical indications for brain MRI examinations: A single-centre study in Ghana	Piersson A.D et al. 2018	No implementation of imaging referral guidelines.
An evaluation of MRI lumbar spine scans within a community-based diagnostic setting	Hudson D et al. 2021	No implementation of imaging referral guidelines.
An investigation of lumbar spine magnetic resonance referrals in two Irish university teaching centres: Radiology clinical judgement versus iRefer guideline compliance	Alanazi, AH et al. 2022	No implementation of imaging referral guidelines.
Applicability of the appropriate use criteria for myocardial perfusion scintigraphy	de Oliveira A et al. 2014	No implementation of imaging referral guidelines.
Application of the ESR iGuide clinical decision support system to the imaging pathway of patients with hepatocellular carcinoma and cholangiocarcinoma: preliminary findings	Gabelloni, M et al. 2020	No implementation of imaging referral guidelines.
Appropriateness and clinical outcome of chest computed tomography without intravenous contrast: A study conducted in Pakistan	Sattar A et al. 2018	No implementation of imaging referral guidelines.
Appropriateness and imaging utilization: "Computerized provider order entry and decision support"	Thrall J.H. 2014	Commentary paper.<br><br>No implementation of imaging referral guidelines.
Appropriateness Criteria for Neuroimaging of Adult Headache Patients in the Emergency Department How Are We Doing?	Heetderks-Fong, E 2019	No implementation of imaging referral guidelines.
Appropriateness of computed tomography and magnetic resonance imaging scans in a rural regional hospital in South Africa: A 6-year follow-up study	Fouche P.E et al. 2021	No implementation of imaging referral guidelines.
Appropriateness of computed tomography and magnetic resonance imaging scans in the Eden and Central Karoo districts of the Western Cape Province, South Africa	Becker J et al. 2014	No implementation of imaging referral guidelines.
Appropriateness of extremity magnetic resonance imaging examinations in an academic emergency department observation unit	Glover M et al. 2018	No implementation of imaging referral guidelines.
Appropriateness of Head CT Scans at Tikur Anbessa Specialized Hospital, Ethiopia	Demeke E. and Mekonnen A. 2022	No implementation of imaging referral guidelines.
Appropriateness of imaging for lung cancer staging in a national cohort	Backhus L.M et al. 2014	No implementation of imaging referral guidelines.

Appropriateness of imaging modality choice by doctors at the Kenyatta National Hospital's Accident and Emergency Department	Ahmed S.S et al. 2022	No implementation of imaging referral guidelines.
Appropriateness of knee MRI prescriptions: clinical, economic and technical issues	Solivetti F.M et al. 2016	No implementation of imaging referral guidelines.
Appropriateness of lumbar spine radiography and factors influencing imaging ordering patterns: paving the path towards value-driven healthcare	Tay Y.X et al. 2023	No implementation of imaging referral guidelines.
Appropriateness of referrals from primary care for lumbar MRI	Krogh S.B et al. 2022	No implementation of imaging referral guidelines.
Appropriateness-based reimbursement of elective invasive coronary procedures in low- and middle-income countries: Preliminary assessment of feasibility in India	Karthikeyan G et al. 2017	No implementation of imaging referral guidelines.
Assessing the Appropriateness of Outpatient Abdominopelvic CT and MRI Examinations Using the American College of Radiology Appropriateness Criteria	Rosenkrantz A.B et al. 2015	No implementation of imaging referral guidelines.
Assessment of Appropriateness of Doing CT Scan for Investigating Headache in a Tertiary Care Hospital in Eastern India	Maitra D et al. 2022	No implementation of imaging referral guidelines.
Assessment of Pediatric Neurotrauma Imaging Appropriateness at a Level I Pediatric Trauma Center	Rao S et al. 2016	No implementation of imaging referral guidelines.
Association Between Physicians' Appropriate Use of Echocardiography and Subsequent Healthcare Use and Outcomes in Patients With Heart Failure	Tharmaratnam, T et al. 2020	No implementation of imaging referral guidelines.
Asymmetric Hearing Loss Prompting MRI Referral in a Military Population: Redefining Audiometric Criteria	Tolisano A.M et al. 2018	No implementation of imaging referral guidelines.
Barriers to collaboration in mental health services for older people: External agency views	Crotty M.M et al. 2014	No implementation of imaging referral guidelines.
Bronchiolitis in children - do we choose wisely?	Kusak B. et al. 2018	No implementation of imaging referral guidelines.
Budget impact of applying appropriateness criteria for myocardial perfusion scintigraphy: The perspective of a developing country	dos Santos M.A et al. 2016	No implementation of imaging referral guidelines.
Cardiovascular Magnetic Resonance Imaging—Incremental Value in a Series of 361 Patients Demonstrating Cost Savings and Clinical Benefits: An Outcome-Based Study	Hegde V.A. et al. 2017	No implementation of imaging referral guidelines.
Choosing radiology imaging modalities to meet patient needs with lower environmental impact	Alshqaqeeq F. et al. 2020	No implementation of imaging referral guidelines.<br><br>No impact/effect on radiology and patient - ocuses on energy costs
Clinical and prognostic value of stress echocardiography appropriateness criteria for evaluation of coronary artery disease in a tertiary referral centre	Bhattacharyya S et al. 2014	No implementation of imaging referral guidelines.
Clinical Decision Support Systems and Medical Imaging	Karami M. 2015	Commentary paper.<br><br>No implementation of imaging referral guidelines.

Clinical decision support: the role of ACR Appropriateness Criteria	Chan S.S. et al. 2019	Commentary paper.<br><br>No implementation of imaging referral guidelines.
Clinical situations for which 3D printing is considered an appropriate representation or extension of data contained in a medical imaging examination: Adult cardiac conditions	Ali A et al. 2020	No implementation of imaging referral guidelines.
Clinical utility and prognostic value of appropriateness criteria in stress echocardiography for the evaluation of valvular heart disease	Bhattacharyya S et al. 2013	No implementation of imaging referral guidelines.
Cost-risk-benefit analysis in diagnostic radiology with special reference to the application of referral guidelines	Moores B.M. 2019	Commentary paper.<br><br>No implementation of imaging referral guidelines.
CT protocols and radiation doses for hematuria and urinary stones: Comparing practices in 20 countries	Gershan V et al. 2020	No implementation of imaging referral guidelines.
CT Utilization: A Case Study in Iran based on ACR Appropriateness Criteria.	Meidani Z et al. 2017	No implementation of imaging referral guidelines.
Deciding why and when to use CT in children: a radiologist's perspective	Frush D.P. 2014	Commentary paper.<br><br>No implementation of imaging referral guidelines.
Delays in imaging diagnosis of acute abdominal pain in the emergency setting	Fruauff A et al. 2022	Commentary paper.<br><br>No implementation of imaging referral guidelines.
DIAGNOSTIC IMAGING IN A DIRECT-ACCESS SPORTS PHYSICAL THERAPY CLINIC: A 2-YEAR RETROSPECTIVE PRACTICE ANALYSIS.	Crowell MS et al. 2016	No implementation of imaging referral guidelines.
Direct access CT for suspicion of brain tumour: an analysis of referral pathways in a population-based patient group	Zienius K et al. 2019	No implementation of imaging referral guidelines.
Effect of intravenous contrast for CT abdomen and pelvis on detection of urgent and non-urgent pathology: can repeat CT within 72 hours be avoided?	Lamoureux, C et al. 2019	No implementation of imaging referral guidelines.
Effective Risk Stratification of Patients on the Basis of Myocardial Perfusion SPECT Is Dependent on Appropriate Patient Selection	Alexander S., Doukky R. 2015	Commentray paper.<br><br>No implementation of imaging referral guidelines.
Emergency Physicians Choose Wisely When Ordering Plain Radiographs for Low Back Pain Patients	Hiranandani, R et al. 2018	No implementation of imaging referral guidelines.
Evaluating the Quality of a Clinical Mobile App for Physicians' CT Scan Ordering Using the MARS Rating Scale	Meidani Z et al. 2022	No implementation of imaging referral guidelines.
Evaluation of Cancer Patients With Suspected Pulmonary Embolism: Performance of the American College of Physicians Guideline	Qdaisat A et al. 2020	No implementation of imaging referral guidelines.
Evaluation of posttreatment follow-up of patients with prostate cancer relative to the American college of radiology's appropriateness criteria	McDonald J.S. et al. 2015	No implementation of imaging referral guidelines.
General anesthesia/sedation requirement influences the way MRI brain scans are ordered in a tertiary pediatric hospital	Sum, MY et al. 2019	No implementation of imaging referral guidelines.

Guideline Adherence for Echocardiographic Follow-Up in Outpatients with at Least Moderate Valvular Disease	Chan R.H et al. 2015	No implementation of imaging referral guidelines.
How artificial intelligence can help us 'Choose Wisely'.	Mehta N et al. 2021	Commentray paper.<br><br>No implementation of imaging referral guidelines.
Impact of Radiologist-Driven Change-Order Requests on Outpatient CT and MRI Examinations	Pourjabbar S et al. 2020	No implementation of imaging referral guidelines.
Inappropriate utilization of SPECT myocardial perfusion imaging on the USA-Mexico border	Lalude, OO et al. 2014	No implementation of imaging referral guidelines.
Inappropriateness of Cardiovascular Radiological Imaging Testing; A Tertiary Care Referral Center Study	Carpeggiani, C et al. 2013	No implementation of imaging referral guidelines.
Inappropriateness of diagnostic imaging examinations in the inpatient setting: a case study research	Squillaci E. et al. 2017	No implementation of imaging referral guidelines.
Incidental findings in emergency imaging: frequency, recommendations, and compliance with consensus guidelines	Hanna T.N et al. 2016	No implementation of imaging referral guidelines.
Indications for Maternal Echocardiography in Detecting Disease and the Impact on Pregnancy Management	Schnettler W.T et al. 2023	No implementation of imaging referral guidelines.
Investigation of Whole Spine MRI in the Emergency Department at Two Large Tertiary Care Academic Medical Centers in the United States	Morris, M et al. 2021	No implementation of imaging referral guidelines.
Justification of CT practices across Europe: results of a survey of national competent authorities and radiology societies	Foley S.J et al. 2022	No implementation of imaging referral guidelines.
Knee MRI Primary Care Ordering Practices for Nontraumatic Knee Pain: Compliance With ACR Appropriateness Criteria and Its Effect on Clinical Management	Gonzalez F.M et al. 2019	No implementation of imaging referral guidelines.
Low back pain in the emergency department - Are the ACR Appropriateness Criteria being followed?	Rao S. et al. 2015	No implementation of imaging referral guidelines.
Magnetic resonance enterography: State of the art	Stoddard P.B. et al. 2015	Commentray paper.<br><br>No implementation of imaging referral guidelines.
Magnitude and financial implications of inappropriate diagnostic imaging for three common clinical conditions	Flaherty, S et al. 2019	No implementation of imaging referral guidelines.
Making the best value of clinical radiology: iRefer Guidelines, 8th edition.	Remedios D et al. 2017	Commentray paper.<br><br>No implementation of imaging referral guidelines.
Measuring appropriateness of diagnostic imaging: a scoping review	Walther F. et al. 2023	No implementation of imaging referral guidelines.
MRI of the knee and shoulder performed before radiography	George E et al. 2014	No implementation of imaging referral guidelines.
Multidetector computed tomography utilization in an urban sub-saharan africa setting: User characteristics, indications and appropriateness	Tambe J et al. 2020	No implementation of imaging referral guidelines.
Multiphase abdomen-pelvis CT in women of childbearing potential (WOCBP) Justification and radiation dose	Al Naemi, H et al. 2020	No implementation of imaging referral guidelines.

National audit of appropriate imaging	Remedios D et al. 2014	No implementation of imaging referral guidelines.
National audit on the appropriateness of CT and MRI examinations in Luxembourg	Bouette, A et al. 2019	No implementation of imaging referral guidelines.
Neuroimaging overuse is more common in Medicare compared with the VA	Burke J.F et al. 2016	No implementation of imaging referral guidelines.
Neuroimaging Wisely	Buethe, J et al. 2016	Commentray paper.<br><br>No implementation of imaging referral guidelines.
Ordering of Diagnostic Imaging by Physical Therapists: A 5-Year Retrospective Practice Analysis	Keil A.P et al. 2019	No implementation of imaging referral guidelines.
Partnering With Your Health System to Select and Implement Clinical Decision Support for Imaging	Jensen J.D. and Durand D.J. 2017	Commentray paper.<br><br>No implementation of imaging referral guidelines.
Prospective cost implications with a clinical decision support system for pediatric emergency head computed tomography	Hayatghaibi S.E et al. 2021	No implementation of imaging referral guidelines.
Reducing Unnecessary Shoulder MRI Examinations Within a Capitated Health Care System: A Potential Role for Shoulder Ultrasound	Sheehan S.E et al. 2016	No implementation of imaging referral guidelines.
Referral criteria and clinical decision support: radiological protection aspects for justification	del Rosario Pérez M. 2015	Commentray paper.<br><br>No implementation of imaging referral guidelines.
Spectrum of echocardiographic abnormalities among 168 consecutive referrals to an urban private hospital in south-western Nigeria	Oyedeji A.T et al. 2014	No implementation of imaging referral guidelines.
Survey of inappropriate use of magnetic resonance imaging.	Oikarinen H et al. 2013	No implementation of imaging referral guidelines.
Systematic assessment of procedural parameters, influence on downstream testing and 12-month outcomes of a CT-myocardial perfusion service	Ho K.-T et al. 2019	No implementation of imaging referral guidelines.
Testing Real-World Application of Appropriateness Criteria of Single Photon Emission Computed Tomography (SPECT) In Two Egyptian Hospitals	Abdeltawab A et al. 2020	No implementation of imaging referral guidelines.
The degree and appropriateness of computed tomography utilization for diagnosis of headaches in Ghana	Gorleku, PN et al. 2021	No implementation of imaging referral guidelines.
The effective and collective dose to patients undergoing abdominopelvic and trunk computed tomography examinations: A Belgian multicentre study	De Roo B et al. 2019	No implementation of imaging referral guidelines.
The use of neuroimaging in dementia by Irish general practitioners	Ciblis A.S et al. 2016	No implementation of imaging referral guidelines.
To what extent do hospitalised patients receive appropriate CT and MRI scans? Results of a cross-sectional study in Southern Italy	Bianco A et al. 2018	No implementation of imaging referral guidelines.
Trends in Hospital Performance on the Medicare National Outpatient Imaging Metrics: A 5-Year Longitudinal Cohort Analysis	Narayan A.K et al. 2019	No implementation of imaging referral guidelines.
Unindicated multiphase CT scans in non-traumatic abdominal emergencies for women of reproductive age: a significant source of unnecessary exposure	Giannitto C et al. 2018	No implementation of imaging referral guidelines.

Unnecessary ordering of magnetic resonance imaging of the knee: A retrospective chart review of referrals to orthopedic surgeons	Mohammed H.T et al. et al. 2020	No implementation of imaging referral guidelines.
Using Digital Health to Support Best Practices: Impact of MRI Ordering Guidelines Embedded Within an Electronic Referral Solution	Huebner L.-A et al. 2019	No implementation of imaging referral guidelines.
Utilization Patterns of Single-Photon Emission Cardiac Tomography Myocardial Perfusion Imaging Studies in a Rural Tertiary Care Setting	Singh, M et al. 2014	No implementation of imaging referral guidelines.
Validation of clinical criteria for referral to head imaging in the neurologic emergency setting	Žužek P et al. 2019	No implementation of imaging referral guidelines.

Supplemental Table 2: Full mixed methods appraisal tool (MMAT) report							
Title	Analysis of Low Appropriateness Score Exam Trends in Decision Support-based Radiology Order Entry System	Improved Appropriateness of Advanced Diagnostic Imaging After Implementation of Clinical Decision Support Mechanism	Justification of CT scans using referral guidelines for imaging	Patients undergoing recurrent CT exams: assessment of patients with non-malignant diseases, reasons for imaging and imaging appropriateness	Screening cervical spine CT in the emergency department, phase 3: Increasing effectiveness of imaging	Use of a Commercially Available Clinical Decision Support Tool to Expedite Prior Authorization in Partnership With a Private Payer	A clinical decision support system to increase appropriateness of diagnostic imaging prescriptions
Author Names	Gupta et al. 2017	Chepelev et al. 2021	Stanescu et al. 2015	Rehani et al. 2020	Griffith et al. 2014	Gaskin et al. 2021	Calcaterra et al. 2018
Category of study design *Based on MMAT	Quantitative descriptive studies	Quantitative descriptive studies	Quantitative descriptive studies	Quantitative descriptive studies	Quantitative descriptive studies	Quantitative descriptive studies	Quantitative descriptive studies
Is the sampling strategy relevant to address the research question?							
Is the sample representative of the target population?							
Are the measurements appropriate?							
Is the risk of nonresponse bias low?							
Is the statistical analysis							

appropriate to answer the research question?							

Yes	No	Unclear

Title	Randomized clinical trial of a clinical decision support tool for improving the appropriateness scores for ordering imaging studies in primary and specialty care ambulatory clinics	Precommitting to choose wisely about low-value services: A stepped wedge cluster randomised trial
Author Names	Palen et al. 2019	Kullgren et al. 2018
Category of study design *Based on MMAT	Randomised controlled trials	Randomised controlled trials
Is randomization appropriately performed?		
Are the groups comparable at baseline?		
Are there complete outcome data?		
Are outcome assessors blinded to the intervention provided?		
Did the participants adhere to the assigned intervention?		

Yes	No	Unclear

Eur Radiol (2024) Tay YX, Foley S, Killeen R et al

Title	Assessment of the Radiology Suppor Communication and Alignment Network to Reduce Medical Imaging Overutilization: A Multipractice Cohort Study	Effect of clinical decision support on appropriateness of advanced imaging use among physicians-in-training	Effectiveness of clinical imaging guidelines to reduce inappropriate head computed tomography imaging: a case of Uganda	Evaluating the Effect of Unstructured Clinical Information on Clinical Decision Support Appropriateness Ratings	Impact of a Commercially Available Clinical Decision Support Program on Provider Ordering Habits
Author Names	Rezaii et al. 2020	Poeran et al. 2019	Kawooya et al. 2022	Moriarity et al. 2017	Huber et al. 2018
Category of study design *Based on MMAT	Non-randomised studies	Non-randomised studies	Non-randomised studies	Non-randomised studies	Non-randomised studies
Are the participants representative of the target population?					
Are measurements appropriate regarding both the outcome and intervention (or exposure)?					
Are there complete outcome data?					
Are the confounders accounted for in the design and analysis?					
During the study period, is the intervention administered (or exposure occurred) as intended?					

Yes	No	Unclear

Title	Reduction in inappropriate MRI knee studies after implementation of an appropriateness checklist: Experience at a tertiary care centre	Reduction in the number and associated costs of unindicated dual-phase head CT examinations after a quality improvement initiative	Teaching cost-conscious medicine: impact of a simple educational intervention on appropriate abdominal imaging at a community-based teaching hospital.	The effect of clinical decision support for advanced inpatient imaging	To Sustain or not to Sustain: Varying Educational Sessions on Advanced Imaging of Low Back Pain and R-SCAN
Author Names	Xu et al. 2020	Strother et al. 2013	Covington et al. 2013	Moriarity et al. 2015	Wang et al. 2021
Category of study design *Based on MMAT	Non-randomised studies	Non-randomised studies	Non-randomised studies	Non-randomised studies	Non-randomised studies
Are the participants representative of the target population?					
Are measurements appropriate regarding both the outcome and intervention (or exposure)?					
Are there complete outcome data?					
Are the confounders accounted for in the design and analysis?					
During the study period, is the intervention administered (or exposure occurred) as intended?					

Yes	No	Unclear

Title	Development of clinical-guideline-based mobile application and its effect on head CT scan utilization in neurology and neurosurgery departments	Effect of governmental intervention on appropriateness of lumbar MRI referrals: A canadian experience	Impact on radiological practice of active guideline implementation of musculoskeletal guideline, as measured over a 12-month period	Justification and active guideline implementation for spine radiography referrals in primary care	Justification of CT examinations in young adults and children can be improved by education, guideline implementation and increased MRI capacity
Author Names	Meidani et al. 2022	Kennedy et al. 2014	Gransjøen et al. 2021	Tahvonen et al. 2017	Tahvonen et al. 2013
Category of study design *Based on MMAT	Non-randomised studies	Non-randomised studies	Non-randomised studies	Non-randomised studies	Non-randomised studies
Are the participants representative of the target population?					
Are measurements appropriate regarding both the outcome and intervention (or exposure)?					
Are there complete outcome data?					
Are the confounders accounted for in the design and analysis?					
During the study period, is the intervention administered (or exposure occurred) as intended?					

Yes	No	Unclear

Title	Predicted Cost Savings Achieved by the Radiology Support, Communication and Alignment Network from Reducing Medical Imaging Overutilization in the Medicare Population	Reducing Inappropriate Lumbar Spine MRI for Low Back Pain: Radiology Support, Communication and Alignment Network	The effect of interventions on appropriate use of lumbar spine radiograph and CT examinations in young adults and children: a three-year follow-up	Impact of the publication of appropriate use criteria on utilization rates of Myocardial perfusion imaging studies in Ontario, Canada: A population-based study	Impact of a radiological protection campaign in emergency paediatric radiology: a multicentric observational study in Brazil
Author Names	Wintermark et al. 2021	Wang et al. 2018	Tahvonen et al. 2020	Roifman et al. 2017	Oliveira et al. 2022
Category of study design *Based on MMAT	Non-randomised studies	Non-randomised studies	Non-randomised studies	Non-randomised studies	Non-randomised studies
Are the participants representative of the target population?					
Are measurements appropriate regarding both the outcome and intervention (or exposure)?					
Are there complete outcome data?					
Are the confounders accounted for in the design and analysis?					
During the study period, is the intervention administered (or exposure occurred) as intended?					

Yes	No	Unclear

Supplemental Table 3: Characteristics of the included studies, outcome and quality assessment result										
**Author (year)**	**Country**	**Methods**	**Clinical Setting**	**Population**	**Imaging Referral Guidelines**	**Mode of Intervention**	**Body Region**	**Imaging Modalities**	**Outcome**	**Quality assessment**
Bhattacharyya and Lloyd (2015) (54)	Not Applicable	Review of evidence	Not Applicable	Not Applicable	ACR Appropriateness Criteria / Choosing Wisely	Application / Education /	Cardiac	CT	1. Integration of information technology tools at the point of referral may aid decision-making and improve efficiency of cardiovascular imaging	Not Applicable
Calcaterra et al. (2018) (46)	Italy	Retrospective data analysis	Hospital / Primary Care	Emergency /Inpatient/ Outpatient	Diagnostic Imaging Referral Guidelines (DIRGs)	Standalone CDSS	Not indicated	Not indicated	1. Appropriateness of imaging procedures<br>2. Procedures prescribed by physicians without the assistance of the CDSS with those prescribed after the advice provided by the CDSS	
Chepelev et al. (2021) (41)	USA	Retrospective data analysis	Not indicated	Not indicated	ACR Appropriateness Criteria	Point-of-care CDSS	Not indicated	CT, MRI, US, NM	1. Proportion of various appropriateness score<br>2. Quantitative relationship between CDSS exposure and imaging appropriateness	*

Covington et al. (2013) (31)	USA	Before-and-after	Hospital	Inpatient	ACR Appropriateness Criteria	Education	Abdomen	CT, MRI, US, Conventional Radiography (CR)	1. Total number of abdominal imaging studies<br>2. Average patient length of stay, in-hospital mortality, average patient radiation exposure from medical imaging, patient satisfaction scores, average patients per inpatient team and average imaging studies completed per patient per hospital day	
Gaskin et al. (2021) (45)	USA	Retrospective data analysis	Hospital	Outpatient	ACR Appropriateness Criteria	Point-of-care CDSS	Not indicated	MRI, CT, PET, NM	1. CDS Scores<br>2. Participation rate in the expedited preauthorisation program	
Gransjøen et al. (2021) (30)	Norway	Interrupted time series	Hospital/ Private Institutions	Outpatient	Norwegian musculoskeletal guideline	Education	Shoulder, Lumbar Spine, Knee	CT, MRI, US, CR	1. Volume of diagnostic imaging of the musculoskeletal system for the four body parts following implementation of the guideline	*
Griffith et al. (2014) (32)	USA	Prospective data analysis	Hospital	Emergency	ACR Appropriat	Education	Cervical Spine	CT	1. Adherence to appropriateness guidelines	*

					eness Criteria				2. Utilisation of imaging services	
Gupta et al. (2017) (37)	USA	Retrospective data analysis	Not indicated	Outpatient	ACR Appropriateness Criteria	Point-of-care CDSS	Extremity, Spine, Cardiac, Face, Sinus, Head CT, Angiography for abdominal aortic aneurysm, Others	CT, MRI	1. Decision support scores<br>2. Report features	*
Huber et al. (2018) (40)	USA	Before-and-after	Hospital	Emergency /Inpatient	ACR Appropriateness Criteria	Point-of-care CDSS	Not indicated	CT, MRI, US, PET, NM	1. Appropriateness of inpatient and emergency imaging procedures<br>2. Performance of trainees and attending physicians<br>3. Performance across imaging modalities	*
Kawooya et al. (2022) (48)	Uganda	Before-and-after	Hospital	Not indicated	ESR iGuide	Application	Head	CT	1. Appropriateness level of imaging procedures<br>2. Knowledge level on radiation protection and clinical imaging guidelines	

Kennedy et al. (2014) (52)	Canada	Before-and-after	Hospital	Not indicated	ACR Appropriateness Criteria	Campaign	Lumbar Spine	MRI	1. Appropriateness of lumbar MRI referrals for low back pain at tertiary care hospitals<br>2. Changes in appropriateness level after enactment of policy changes<br>3. Changes in the number of new lumbar MRI referrals after the policy change	
Kullgren et al. (2018) (53)	USA	Stepped wedge, cluster randomised clinical trial	Primary Care clinics	Outpatient	Choosing Wisely	Campaign	Head, Lumbar Spine	CT, MRI, CR	1. Difference between the control and intervention periods in the percentage of visits with an applicable potentially low-value order: a lumbar spine X-ray, CT or MRI order in visits for low back pain; a head CT or MRI order in visits for headaches<br>2. difference in these percentages between the control and follow-up periods to assess whether any intervention effect	

									was sustained in the short term following the intervention	
Meidani et al. (2022) (47)	Iran	Before-and-after	Hospital	Not indicated	Care Core Criteria for Imaging	Application	Head	CT	1. CT scan utilisation per patients<br>2. Total head CT utilisation	
Moriarity et al. (2015) (39)	USA	Before-and-after	Hospital	Inpatient	ACR Appropriateness Criteria	Point-of-care CDSS	Not indicated	CT, MRI, NM	1. Overall imaging-request appropriateness<br>2. Corresponding ACR AC scores generated when requesting advanced imaging	
Moriarity et al. (2017) (44)	USA	Before-and-after	Hospital	Inpatient	ACR Appropriateness Criteria	Point-of-care CDSS	Not indicated	CT, MRI, PET, NM	1. Average appropriateness rating<br>2. Percentage of requests without enough information to generate appropriateness rating	
Oliveira et al. (2022) (27)	Brazil	Before-and-after	Private healthcare-associated unit	Emergency	Image Gently / ACR Appropriateness Criteria / RCR iRefer / ESR iGuide	Education	Paranasal sinus, Chest, Others	CT, CR	1. Changes in the referrals of imaging procedures in a paediatric population	

Palen et al. (2019) (43)	USA	Stepped wedge, cluster randomized clinical trial	Integrated healthcare delivery system	Outpatient	ACR Appropriateness Criteria	Point-of-care CDSS	Not indicated	CT, MRI	1. The proportion of ACR Select appropriateness scores of 7 or greater before and after activation of the CDSS<br>2. The rate per month of advanced imaging orders before and after activation of the CDSS<br>3. The proportion of orders in which the radiology department requested changes	
Poeran et al. (2019) (38)	USA	Before-and-after	Hospital	Emergency	ACR Appropriateness Criteria	Point-of-care CDSS	Abdomen, Head, Chest, Others	CT, MRI	1. Appropriateness scores of imaging procedures<br>2. Percentage of appropriate referrals (4-9)	*
Rehani et al. (2020) (42)	USA	Retrospective data analysis	Hospital	Not indicated	ACR Appropriateness Criteria	Point-of-care CDSS	Not indicated	CT	1. Rate of CT referrals meeting accepted criteria for imaging	*
Rezaii et al. (2020) (49)	USA	Before-and-after	Private and academic practices	Not indicated	ACR Appropriateness Criteria / Choosing Wisely	Collaboration and communication	Chest, Pelvis (Adnexal Cyst), Lumbar	CT, MRI	1. Proportion of high-value imaging cases<br>2. Total number of low-value imaging cases before and after intervention	*

							Spine, Others			
Roifman et al. (2017) (35)	Canada	Retrospective cohort	Not indicated	Not indicated	Appropriate Use Criteria	Guidelines dissemination	Cardiac	CT	1. Changes in utilisation rate<br>2. Cost savings	*
Sheng et al. (2016) (55)	Not Applicable	Review of evidence	Not Applicable	Not Applicable	ACR Appropriateness Criteria	Education	Not indicated	Not indicated	1. Educational interventions increase awareness of the appropriateness criteria as a decision tool	Not Applicable
Stanescu et al. (2015) (36)	Romania	Retrospective data analysis	Hospital	Emergency	National guidelines (Good Practice Guide)	Guidelines dissemination	Abdomen, Pelvis, Spine, Cranial, Mastoid, Limbs, Thorax, Urological	CT	1. Conformity rate with Good Practice Guide<br>2. Total collective dose	
Strother et al. (2013) (33)	USA	Before-and-after	Hospital	Outpatient/ Inpatient	ACR Appropriateness Criteria	Education	Head	CT	1. The appropriateness scores of the dual-phase head CT examination performed before and after the intervention<br>2. Changes in the number of dual-phase head CT	

									examinations performed<br>3. Differences in costs that were associated with the intervention	
Tahvonen et al. (2013) (28)	Finland	Before-and-after	Hospital	Outpatient	European Commission. Radiation protection 118	Education	Head, Thorax, Lumbar Spine, Abdomen or upper abdomen, Trauma, Cervical Spine, Nasal Sinuses, Body (Thorax and abdomen), Others	CT	1. Changes to the total number of CT examination performed<br>2. Rate of justification of various CT examination	
Tahvonen et al. (2017) (29)	Finland	Before-and-after	Hospital	Not indicated	European Commission. Radiation protection 118	Education	Cervical Spine, Thoracic Spine, Lumbar Spine	CR	1. Changes in the volume of practitioners' requests for spine radiographs before and after intervention<br>2. Changes in the volume of examination in	

									accordance with the guidelines before and after intervention<br>3. Changes in referrals to MRI examination<br>4. Changes in collective effective dose	
Tahvonen et al. (2020) (26)	Finland	Before-and-after	Hospital	Not indicated	European Commission. Radiation protection 118	Education	Lumbar Spine	CT, CR	1. Changes in the volume of lumbar spine CT and radiography examination after intervention<br>2. Appropriateness of lumbar spine CT and radiography examination	
Wang et al. (2018) (51)	USA	Before-and-after	Family Medicine Clinics	Outpatient	ACR Appropriateness Criteria / Choosing Wisely	Collaboration and communication	Lumbar Spine	MRI	1. Appropriateness rating of visitation during the 4-month preeducation and 3-month posteducation periods<br>2. Changes in the number of physical therapy referrals during the 4-month preeducation and 3-month posteducation periods	

									3. Changes in the time from initial clinic visit for uncomplicated LBP to receiving an LS MRI exam<br>4. Changes in the number of LS MRI referrals for LBP made per month per clinical during the study period	
Wang et al. (2021) (50)	USA	Before-and-after	Primary Care	Outpatient	ACR Appropriateness Criteria / Choosing Wisely	Collaboration and communication	Lumbar Spine	MRI	1. Appropriateness rating for examination<br>2. Changes in number of MRI orders per clinical per month<br>3. Changes in physical therapy referrals before and after intervention	
Wintermark et al. (2021) (13)	USA	Before-and-after	Not indicated	Not indicated	ACR Appropriateness Criteria / Choosing Wisely	Collaboration and communication	Chest, Pelvis (Adnexal Cyst), Lumbar Spine	CT, MRI	1. Proportion of high-value cases<br>2. Cost savings	*
Xu et al. (2020) (34)	Canada	Before-and-after	Hospital	Not indicated	ACR Appropriateness Criteria	Checklist	Knee	MRI	1. Rate of knee MRI showing moderate or greater OA	

									2. The absolute and relative number of knee MRIs done	
*These refer to studies that satisfy all the specified criteria in the mixed methods appraisal tool (MMAT)										

Supplemental Table 4: Key findings and conclusion of the included studies		
**Author (year)**	**Findings**	**Conclusion**
Bhattacharyya and Lloyd (2015) (54)	1. Data suggest that publication of appropriateness criteria has not had a universal effect on improving appropriateness of all imaging modalities.<br>2. Time required to review each imaging request with the guidelines may pose problems of sustainability of long-term use and applicability to daily clinical practice.<br>3. A sustained program of regular education and feedback may be required to sustain the reduction in inappropriate testing.<br>4. The use of a multifaceted approach, including individual feedback, is key.	Engagement of stakeholders, including referring physicians, imagers, and patients, is essential to reduce inappropriate testing. Educational interventions to improve accuracy should include review of reference cases and individual, personalised feedback in addition to didactic training. Integration of information technology tools at the point of referral, at reporting, and for quality assurance may aid decision-making and improve efficiency.
Calcaterra et al. (2018) (46)	1. 23% of cases the physician indicated some exams which eventually turned out to be inappropriate according to the DIRGs.<br>2. After the CDSS displayed the list of potential appropriate exams, a reduction of about 20% in the number of inappropriate prescriptions is evident with a 9.5% increase in the first choice. (Proposed 3 choices)<br>3. With the guidance of the CDSS, a reduction of about 15% in the number of inappropriate prescriptions is observed. (Proposed 4 choices)	The system went through broad approval tests and the experimental findings corroborate its effectiveness in drastically lowering inappropriate prescriptions.
Chepelev et al. (2021) (41)	1. In the observational data: the green rate varied from 50.7% in the lowest chronological bin index to 57.5% in bin 20, the yellow rate from 18.1 to 14.8%, the red rate from 13.8 to 12.6%, and the total number of requisitions from 1,554,847 to 104,997.<br>2. In the first analysis, the fraction of green requisitions increased from 54.5 to 57.5% with increasing CDS exposure, the yellow requisitions decreased from 15.5 to 14.8%, and the red requisitions decreased 15.6% to 12.6%. Thus, the fraction of green, yellow, and red requisitions among the last 10 requisitions changed by +3.0%, −0.8%, and −3.0% in comparison with the first 10, respectively.<br>3. Providers with > 190 requisitions had 8.5% more green requisitions, 2.3% fewer yellow requisitions, and 0.5% fewer red (requisitions relative to providers with ≤ 10 requisitions.	The positive association of increased CDSM exposure with improved requisition appropriateness scores supports CDSM consultation for high cost imaging.
Covington et al. (2013) (31)	1. Total abdominal imaging studies per patient (all imaging modalities) also were significantly reduced from 3.1 to 2.7 studies.<br>2. Abdominal CT scans per patient were significantly reduced from 1.7 to 1.4 studies.	A simple educational intervention on cost-conscious medicine appeared to reduce radiologic test-ordering behavior of internal medicine

	3. Combined MRI and ultrasound studies per patient (radiation-free modalities) showed no significant difference (0.60–0.57 studies, respectively.<br>4. The mean number of abdominal CT scans per patient per hospital day decreased significantly from 0.50 studies to 0.43 studies. Combined MRI and ultrasound studies per patient per hospital day was unchanged at 0.18 studies. The total radiology studies per patient per hospital day (all modalities) and the average patient length of stay demonstrated no significant difference (0.80– 0.75 studies per patient per day.<br>5. There was no difference for in-hospital mortality pre and post intervention (2.17% and 1.70%, respectively. The average patient radiation dose from medical imaging decreased significantly from 16.7 mSv to 14.0 mSv. There was no significant difference in the average number of patients per inpatient team (12.53 for pre- and 13.53 for postintervention periods.<br>6. Patient satisfaction data - The proportion of patients who rated their overall medical care as 9 or greater on a 10-point survey (10 signifying excellence) was not significantly different between the 2 years (69% and 68%, respectively).<br>7. The avoidance of charges attributed to the intervention from abdominal CT scans alone was US$ 129 per patient or US$ 81,528 in total. This is significant because the intervention was completed at minimal cost (total costs are estimated at US$ 3,605).	residents on the inpatient floors. Widespread adoption of similar educational interventions by other residency programs could result in significant savings to hospitals and the health care system.
Gaskin et al. (2021) (45)	1. 1,453 advanced outpatient imaging orders for Aetna members were placed with structured indications, allowing for CDS scores to be generated. Of these CDS-scored orders, 997 (69%) received scores of 7 to 9 and underwent expedited prior authorization.<br>2. As a result of CDS being optional, many ordering providers continued to enter orders with only free text reasons for examination, yielding no CDS score for these orders. The lack of a CDS score rendered 8,187 of 9,640 (85%) orders ineligible for the pilot program.	In collaboration with a private payer and an RBM, an AMC employed a commercially available CDS order entry tool for advanced imaging tests and was able to avoid the traditional prior authorization process in 69% of orders with CDS scores. Orders with high CDS scores (ie, 7-9) went through a new process of expedited prior authorization.
Gransjøen et al. (2021) (30)	1. There is a statistically significant level change for all four examinations most likely to contain a high degree of unwarranted examinations (MRI shoulder and knee, and X-ray lower back and shoulder).<br>2. The level change seen here equates to a reduction in the use of these examinations of 62.8 examinations per 105, per month, which is an average reduction of 11.9% the first year after the intervention ($p = 0.05$).<br>3. A reduction of 70.5 examinations per 105 per month, which corresponds to an average reduction of 23.4% per month the forts year after the intervention ($p = 0.002$), was found for	The impact of a multifaceted implementation of musculoskeletal referral guidelines on the use of diagnostic imaging of the neck, shoulder, lower back, and knee is uncertain. There was found a significant reduction in the use of MRI examinations deemed most likely to have a great portion of unwarranted imaging in the intervention county, indicating a reduction in unwarranted imaging.

	the use of MRI examinations previously shown to contain a high rate of unwarranted examinations (MRI shoulder and knee) for the intervention county.	
Griffith et al. (2014) (32)	1. Of the 376 examinations with no acute injuries, 49 (13%) met all five NEXUS criteria, compared with 16.1% in phase 2. In addition, 312 had no documentation of altered level of consciousness, thus making the patients eligible for screening with the CCR criteria. Of these 312, 71 patients (22.8%) had none of the abbreviated CCR criteria and should not have undergone imaging. Fifteen of the 376 patients (4%) without acute injuries required no imaging when both the NEXUS and abbreviated CCR criteria were appropriately applied. This was significantly decreased from 7.6% in phase 2 (P = .027).<br>2. The reduction in the rate of overutilization according to the NEXUS criteria between phases 2 and 3 (from 16.1% to 13%) was not statistically significant. When allowing for application of either the NEXUS or abbreviated CCR criteria, the reduction in rate of overutilization from 7.6% in phase 2 to 4.0% in phase 3 was statistically significant.<br>3. The reduced rates of overutilization, in addition to the increase in rate of positive studies between phase 2 (1.0%) and phase 3 (3.1%), point to improved clinical effectiveness of imaging with stricter application of appropriateness criteria, an expected result	Clinical education initiatives such as those in this study have the potential to improve adherence to evidence based clinical guidelines, such as the ACR Appropriateness Criteria, and, in doing so, increase the clinical effectiveness of imaging examinations.
Gupta et al. (2017) (37)	1. Low clinical appropriateness score 0-3 (n=12,615/445,984; 2.8%).<br>2. Intermediate CAS (n=32,565/445,984; 7.3%).<br>3. High CAS (n=400,804/445,984; 89.8%).<br>4. Imaging outcome was found to be highest for extremity MRI cases (66.7%). The proportion of exams with positive findings was less for the other patient-demanded exams ranging from 27% for sinus CT to 10.5% for spine CT/MRI. Exams performed for abdominal aortic aneurysm evaluation had the lowest percentage of positive findings (4%).	The implementation of a decision support–enabled order entry system can provide useful guidelines for physicians for placing the most appropriate imaging order; however, it needs to accommodate the relatively few cases where physician insight or other clinical information merits performing an apparently low appropriate score exam.
Huber et al. (2018) (40)	1. Pre-implementation (11% were low utility, 24.5% were marginal utility, and 64.5% were indicated); Post-implementation (5.4% were low utility, 12.6% were marginal utility, and 82% were indicated).<br>2. Comparing the pre-intervention period (ie, silent mode) to the postintervention period (ie, feedback mode), the percentage of low utility studies ordered by attending physicians decreased from 10% to 6.7%, and the percentage of indicated studies rose from 65.3% to 75.7%.	After implementation of a commercially available CDS program, there was a significant improvement in the appropriateness scores of ordered imaging studies.

	3. Similarly, the percentage of low utility studies ordered by trainees (residents or fellows) decreased from 10.8% to 4.8% (95% CI: 4.4%, 5.2%), and the percentage of indicated studies rose from 65.6% to 83.7%.<br>4. Likewise, the percentage of low utility studies ordered by midlevel providers (ie, physician assistants and nurse practitioners) decreased from 13.8% to 10.2%, and the percentage of indicated studies rose from 55.7% to 72.2%<br>5. Comparing the pre-intervention period (ie, silent mode) to the postintervention period (ie, feedback mode), the percentage of low-utility CT studies decreased from 7.4% to 2.5% and the percentage of indicated studies increased from 72.2% to 87.7%.<br>6. Similarly, the percentage of low-utility MRI studies decreased from 19% to 7.4%, and the percentage of indicated studies increased from 59.8% to 73.1%.<br>7. Likewise, the percentage of low-utility US studies decreased from 11.3% to 3.8%, and the percentage of indicated studies increased from 62.1% to 83.2%.<br>8. The percentage of low utility PET and NM studies unexpectedly increased from 15.3% to 29.4%, and the percentage of indicated studies declined slightly from 46.8% to 46.0%.	
Kawooya et al. (2022) (48)	1. Pre-intervention: 53% inappropriateness (n=262) vs Post-intervention: 47% inappropriate (n=154)<br>2. There was a 73% level of knowledge pre-intervention assessment and over 93% level post-intervention CIGs among the referring clinicians.	CIGs are effective to reduce inappropriate brain CT imaging requisitions.
Kennedy et al. (2014) (52)	1. Before the policy change, the percentage of lumbar MRI referrals deemed appropriate was 50.4%, and the percentage deemed not appropriate was 47.9%; 1.7% of referrals were classified as may be appropriate.<br>2. After the policy change, appropriateness seemed to have increased, with 62.6% of referrals being appropriate and 37.1%<br>not appropriate; 0.3% of referrals were classified as may be appropriate.<br>3. The overall mean appropriateness score before the policy change was 5.08. After the policy change, the mean appropriateness score increased significantly to 5.79.	The interventions of the government of Ontario, including guideline and clinical decision-aid distributions, seem to have significantly increased the appropriateness of lumbar MRI referrals. However, the overall appropriateness increase is small, and a large number of referrals continue to be inappropriate. Additionally, no significant decrease in the number of new lumbar MRI referrals has occurred since the policy change.
Kullgren et al. (2018) (53)	1. For low back pain there was a statistically significant decrease in the percentage of visits with an order for a potentially low-value service (−1.2%, 95% CI −2.0% to −0.5%; p=0.001).<br>2. There was no statistically significant decrease in the overall percentage of visits with an order for a potentially low-value service (−1.4%, 95% CI −2.9% to 0.1%; p=0.06) between control and intervention.	A behavioural economic strategy of asking clinicians to precommit to specific Choosing Wisely recommendations paired with decision supports, although theoretically promising and highly scalable, only yielded a small and

	3. A decrease in the percentage of visits for low back pain with an order for a potentially low-value service, the magnitude of this change was small (just 12 fewer potentially low-value orders per 1,000 visits) and not sustained in the near term after the intervention ended.	unsustained decrease in potentially low-value orders for one of three targeted conditions. These results highlight the potential of behavioural economic strategies to be integrated into clinical workflows with minimal resources as well as some of the important challenges to be confronted in interventions to reduce ordering of low-value services.
Meidani et al. (2022) (47)	1. The median for CT scan utilization during three phases of study was 2, third quartiles (Q3) indicate CT scan utilization declines from median= 4 at the before intervention phase to 2 after the intervention phase. This decline (Q3=2) remained the same during the intervention phase and post-intervention follow up phase.<br>2. The decrease of total CT scan utilization at three phases of study at both neurology and neurosurgery departments was statistically significant (P = 0.027).	The effect of guideline -based mobile application was not a long-lasting change, it did not necessarily confirm any cause and effect relationship between the mobile app and physicians' behavior.
Moriarity et al. (2015) (39)	1. The majority of these requests, 76.0% and 81.7% during the baseline and intervention periods, respectively, were considered "appropriate".<br>2. A slight, but statistically significant, increase was seen in the average AC score of all requests, from 7.2 at baseline to 7.4 during the intervention period.<br>3. NM AC Score (6.6 vs 7.3).<br>4. CT AC Score (7.2 vs 7.3).<br>5. MRI AC Score (7.4 vs 7.4).	Integrating CDS into inpatient CPOE slightly increased the overall AC score of advanced imaging requests as well as the provision of sufficient structured data to automatically generate AC scores. Both effects were more pronounced in PCPs compared with specialists.
Moriarity et al. (2017) (44)	1. There was a statistically significant increase in the overall request AR by CDS alone during the intervention (P = 0.001), which was reduced when comparing the AR with radiologist review between periods but remained statistically significant (P = 0.022).<br>2. Unrated requests were overwhelmingly due to insufficient structured data provided for CDS to generate an AR (73.9% during baseline and 70.7% during intervention), rather than the lack of applicable ACR AC (0.7% during baseline and 0.9% during intervention).	Despite limited effective use of CDS by providers during the study, CDS was associated with a slightly increased percentage of requests rated "appropriate" and an increase in the percentage of requests containing additional, relevant clinical information. When the provided clinical information was reviewed, the majority of advanced inpatient imaging requests had overall high rates of appropriateness by currently available ACR AC. Compared with the prospective AR by CDS software in isolation, incorporating unstructured clinical information significantly

		increased the overall appropriateness of advanced inpatient imaging requests.
Oliveira et al. (2022) (27)	1. In the 12 months after the campaign, there was a reduction in the radiological exams at paediatric emergencies in the engaged units.<br>2. These reductions represented an overall reduction of 12,906 (25%) in the number of referrals for radiological exams in the attendances performed, after justification and implementation of the radioprotection campaign.	The campaign resulted in a substantial reduction in radiological referrals while promoting a radiation protection culture in the department. Simple education initiatives can contribute to both financial and radiation doses savings, particularly important in radiosensitive cohorts. Continuous education is especially important to change the culture of overuse, as well as information and communication to patients and carers about the benefits and risks of ionising radiation.
Palen et al. (2019) (43)	1. When the CDS BPA was inactive, clinicians had an adjusted proportion of appropriateness scores of 7 or greater of 77.0%, which increased to 80.1% after CDS BPA activation.<br>2. The rate of imaging orders did not change significantly after CDS BPA activation (The slope for the rate of imaging orders per month over time before CDS BPA activation was 0.092 vs the slope in the subsequent period was 0.035) over time before CDS BPA activation was 0.092.<br>3. The mean number of imaging orders was 10.85 per 1000 member-months before CDS BPA activation (September 2014–September 2015) and 11.14 after activation (October 2015–August 2017).<br>4. The proportion of change order requests made by radiologists to clinicians decreased from 5.7% in the 12 months before CDS implementation to 5.3% in the 12 months after CDS implementation.	CDS systems hold promise to improve the quality of image ordering in clinical practice and may have impacts on patient outcomes and organizational efficiency that deserve further investigation.
Poeran et al. (2019) (38)	1. The three most commonly selected indications were abdominal pain, headache, and suspected pulmonary embolism. Scores increased for abdominal pain, headache, suspected pulmonary embolism, and other indications between the pre-CDS and post-CDS 2 periods.<br>2. Pre-CDS vs Post-CDS 1 (no changes - overall mean score 6.2).<br>3. Post-CDS 2: mean score of 6.7.<br>4. Pre-CDS vs Post-CDS 1: 18.9% vs 19.1%; Post-CDS 2: 10.2% inappropriate orders.	Positive effect of CDS tools in increasing the rate of appropriate imaging use. CDS tools may thus serve as an equalizer in directing quality patient care from physicians across the training spectrum with varying familiarity with Appropriateness Criteria.
Rehani et al. (2020) (42)	1. For all CT exams combined, 2% were red (low utility), 38% were yellow (marginal), 27% were green (indicated), and 33% were unscored.	The institutions where CDS is not in use and where optimization in CT dose is not as good as at our institution are likely to have much more magnitude of at-risk patients. It also appears that while

		appropriateness criteria are available for initial work-up and diagnosis, there is a lack of guidance on serial imaging.
Rezaii et al. (2020) (49)	1. Proportion of high-value cases improved from the baseline phase (57%) to the post educational phase (79%).<br>2. The change in the proportion of high-value cases increased from 55%, 79%, and 48% in the baseline phase to 73%, 91%, and 85%, respectively, in the post educational phase for the CTA for pulmonary embolism, adnexal cyst follow-up, and advanced imaging for low back pain cohorts.<br>3. Total decrease of 568 medium- and low-value cases, of which 345 were low value.	R-SCAN participation resulted in greater proportions of high-value imaging cases as well as reduced numbers of low value imaging cases and thus contributed to the overall goals of the TCPI effort, which includes strengthening the quality of patient care, promoting wise use of health care resources, and moving clinicians into new payment model opportunities.
Roifman et al. (2017) (35)	1. In the time series analysis, we found that the second intervention (publication of the 2009 AUC) was associated with a significant reduction in Myocardial perfusion imaging (MPI) rates after accounting for the background trend, seasonality, and autocorrelation ($P<0.001$). In contrast, publication of the first ($P=0.69$) and third ($P=0.76$) AUC interventions were not associated with a significant reduction in the rate of MPI utilization.<br>2. The reduced utilization rate translated into ≈88 849 fewer MPI scans performed after publication of the 2009 AUC. In addition, total cost reductions were estimated at CAN$ 72,056,539 during that time frame.	Publication of the 2009 AUC was associated with a significant reduction in the MPI utilization rates in Ontario after accounting for background trend and seasonality. This reduced rate translated into ≈88 849 fewer MPI scans performed at a cost savings of ≈72 million Canadian dollars. These population-based results illustrate a potential real-world impact of the MPI AUC.
Sheng et al. (2016) (55)	1. Low utilization of the Appropriateness Criteria (AC) by clinicians when ordering studies.<br>2. Lack of awareness of the AC in undergraduate and graduate medical education.<br>3. Lack of training in imaging utilisation guidelines among non radiology residents.<br>4. Several studies evaluating educational interventions targeting the use of the AC have shown that increasing awareness may improve utilization.	The scarcity of current literature suggests that the rate of awareness and incorporation of the AC into clinical practice is low. This likely reflects the lack of formal training in appropriate imaging ordering practices in both undergraduate and graduate medical education, which may be a result of insufficient nonradiology specialty acceptance.
Stanescu et al. (2015) (36)	1. The conformity with the Guide was assessed to be positive for 67% of all examinations.<br>2. The collective dose for all exams (252 analysed cases) was estimated to be 1,357 man mSv. For examinations non-conforming to the recommendations, the estimated collective dose was 352 man mSv, which represents 26 % of the total collective dose.	The easiest way to improve the justification process of the CT scans at the individual level and to reduce the unnecessary exposure is the dissemination of provisions of the good practice guide by means of training courses for medical doctors, both practitioners and referrers.

Strother et al. (2013) (33)	1. The overall distribution of studies across the four ordinal categories (0 = not rated or not indicated and the three ACR Appropriateness Criteria rating interpretations) did not differ after the initiative.<br>2. There was a statistically significant reduction (p = 0.006) in the number of dual-phase head CT examinations performed after the quality improvement initiative.<br>3. The median number of dual-phase head CT examinations declined from 40 per month in phase I to 17 per month in phase III.<br>4. Before the quality improvement initiative, the average additional health care costs accrued when performing an unindicated dual-phase head CT examination compared with an indicated single-phase head CT examination was US$ 15,564 per month. After the quality improvement initiative, the additional cost fell to an average of US$ 5,775 per month. This savings projects to an estimated reduction in health care costs of US$ 117,000 per year at our institution.<br>5. Decreased volume resulted in improvements in radiation exposure and unnecessary IV contrast administration as well as reduced health care costs.	Incorporating the ACR Appropriateness Criteria applies evidence based medicine to this algorithm. In this outcomes- driven study, the number of unindicated dual-phase head CT examinations was reduced and imaging efficacy was improved primarily through physician education and monitoring.
Tahvonen et al. (2013) (28)	1. The total number of CT examinations increased by 12% (16 975 and 19 046, respectively).<br>2. In the group of patients aged <35 years, the number of lumbar CTs decreased by 79% (p<0.001), cervical spine CTs by 48% (p<0.001) and head CTs by 21% (p<0.001).<br>3. In 2009, 87% of the 177 analysed examinations were justified compared with 71% in 2005 (p<0.001).<br>4. The proportion of justified lumbar CT examinations increased from 23% to 81% (p<0.001). In the abdominal CT group, the proportion of justified cases increased from 63% to 80%. In the cervical spine CT group, the justification remained unchanged.<br>5. The proportion of justified CT examinations remained nearly constant from 2005 (21 children) to 2009, being 86% and 92%, respectively.	It is possible to reduce the number of various CT examinations and to improve their justification in young patients by regular education, guideline implementation and increased MRI capacity.
Tahvonen et al. (2017) (29)	1. The number of different spine radiographs decreased significantly in the 6-month periods in 2011 and 2012 compared with 2010. Spine radiography reduced by 51% from 2010-2012: Cervical reduced by 39%, Thoracic reduced by 63% and lumbar reduced by 53%.<br>2. In 2010, 24% of cervical spine, 46% of thoracic spine, and 32% of lumbar spine radiography was justified.	This study demonstrates that the number of spine radiography examinations in primary care can be reduced significantly by active referral guideline distribution and educational lectures. The results of the study persisted after 1-year follow-up. This reduction was achieved without an increase in referrals to MRI examinations.

	3. The number of spine MRI examinations performed on Oulu residents at the Oulu University Hospital decreased by 8% from 2010 to 2012. MRI cervical: +7.9%; Thoracic: -30.3% and Lumbar: -11.3%.<br>4. The collective effective dose reduction due to the decrease in spine radiography from 2010 to 2012 was 807 mSv (–52%) in the 6-month study period.	
Tahvonen et al. (2020) (26)	1. The volume of lumbar spine radiographs performed on patients aged < 35 years and also on all age groups decreased significantly (> 30%) during the study period.<br>2. The number of lumbar spine CT scans decreased significantly from 2005 to 2007 and the result persisted during the follow-up.<br>3. Overall appropriateness in the units improved from 65% in 2005 to 85% in 2009 ($p = 0.005$).<br>4. The proportion of appropriate lumbar spine CT scans was low in 2005 compared to the other years ($p < 0.001$). The improvement from 2005 to 2007 was already significant ($p = 0.004$) and the level remained unchanged during the follow-up.	A combination of interventions— guideline implementation and education—can achieve a sustained reduction in the number of lumbar spine radiographs and CT scans in young patients and improve appropriateness of the examinations performed.
Wang et al. (2018) (51)	1. The combined average rating for MRIs made at all three clinics was 5.8 after educational sessions, which was significantly higher than the rating of 4.7 before educational sessions (p = .014).<br>2. No significant differences were found in the proportion of physical therapy referrals made by providers for LBP or the proportion of patients who attended a physical therapy appointment before or after educational sessions.<br>3. The duration of time from when the patient initially saw a provider for LBP to the time when the patient ultimately received an LS MRI was significantly longer after educational sessions for each clinic (p = .015 to .046). When assessing the combined average elapsed time from all three clinics, the time to MRI exam was 13.6 weeks during the pre-education period and 21.6 weeks during the post-education period (p = .005).<br>4. During the 10-month pre-education period, a combined 300 LS MRIs were ordered from the three clinics, which is higher than the combined 187 LS MRIs ordered during the 10-month post-education period.	Our institutional experience with R-SCAN demonstrated a reduction in LS MRI studies performed for uncomplicated LBP and improved appropriateness of those studies as measured by the ACR Appropriateness Criteria rating over a 2-year period in a county clinic family medicine setting.
Wang et al. (2021) (50)	1. The ACR Appropriateness Criteria ratings were significantly higher than the baseline ratings obtained before the first education for Clinic C and the combined total. There was a trend toward higher ratings for Clinic B when compared to baseline ratings obtained before the first education.<br>2. There is an overall qualitatively downward trend in the monthly MRI referrals from before the first education to after the second education.	R-SCAN participation offers many advantages to radiologists and their referrers, in addition to the collaborative and patient-centric benefits. R-SCAN may be used as a stop-gap until other mechanisms, such as CDS, can be fully integrated into daily operations.

	3. There were no significant differences in the monthly MRI referral rates, ACR Appropriateness Criteria ratings, or physical therapy referral rates before and after the second education for any of the three clinics.	
Wintermark et al. (2021) (13)	1. R-SCAN intervention led to an increase in the proportion of high-value cases from 55%, 48%, and 79% in the baseline phase to 73%, 85%, and 91%, respectively, in the post-educational phase for the CTA for pulmonary embolism, advanced imaging for low back pain, and adnexal cyst imaging follow-up cohorts.<br>2. Cost saving for CTA: costs decreased from US$ 232,014 in the baseline phase to US$ 127,955 in the post-educational phase (savings of US$ 104,059).<br>3. Cost saving for advanced imaging for lower back pain: the cost of lesser value imaging decreased from US$ 181,144 to US$ 48,305 (savings of US$ 132,839).<br>4. Cost saving for adnexal cyst imaging: the cost of lesser value imaging decreased from US$ 38,034 to US$ 17,088 (savings of US$ 20,946).	Our analysis of the impact of R-SCAN on health care imaging costs shows the potential for substantial savings if the changes in imaging ordering observed in the previously published R-SCAN study are expanded globally for the Medicare population.
Xu et al. (2020) (34)	1. The total number of studies showing moderate or greater grade osteoarthritis (OA) decreased from 73 pre-checklist to 41 post-checklist, a 44 % decrease.<br>2. Pre -and post-checklist studies with moderate-severe grade or greater decreased from 51 to 15 patients respectively, a 71 % decrease and those with severe grade OA decreased from 35 to 7 patients respectively, an 80 % decrease.<br>3. Pre-checklist, 652 MRI knee MRIs were performed, and post-checklist 336 studies were performed, a 48 % decrease.<br>4. Wait times for MRI knee studies decreased from an average of 23.3 ± 9.1 days pre-checklist to 17.4 ± 5.3 days post-checklist.	Our study provides provocative, but early and incomplete, evidence that appropriateness checklists, when enforced with reasonable diligence, can decrease the likelihood of referrals for inappropriate knee MRIs.
